# Automated measurement of bone scan index from a whole-body bone scintigram

**DOI:** 10.1007/s11548-019-02105-x

**Published:** 2019-12-13

**Authors:** Akinobu Shimizu, Hayato Wakabayashi, Takumi Kanamori, Atsushi Saito, Kazuhiro Nishikawa, Hiromitsu Daisaki, Shigeaki Higashiyama, Joji Kawabe

**Affiliations:** 1 Institute of Engineering, Tokyo University of Agriculture and Technology, 2-24-16 Naka-cho Koganei, Tokyo, 184-0012 Japan; 2 Nihon Medi-Physics Co., Ltd, 3-4-10 Shinsuna Koto-ku, Tokyo, 136-0075 Japan; 3 Department of Radiological Technology, Gunma Prefectural College of Health Sciences, 323-1 Kamioki-machi Maebashi, Gunma, 371-0052 Japan; 4 Department of Nuclear Medicine, Graduate School of Medicine, Osaka City University, 1-4-3 Asahimachi Abeno-ku, Osaka, 545-8585 Japan

**Keywords:** Computer-aided interpretation, Deep learning, Bone scintigram, Bone metastatic lesion, Bone scan index

## Abstract

**Purpose:**

We propose a deep learning-based image interpretation system for skeleton segmentation and extraction of hot spots of bone metastatic lesion from a whole-body bone scintigram followed by automated measurement of a bone scan index (BSI), which will be clinically useful.

**Methods:**

The proposed system employs butterfly-type networks (BtrflyNets) for skeleton segmentation and extraction of hot spots of bone metastatic lesions, in which a pair of anterior and posterior images are processed simultaneously. BSI is then measured using the segmented bones and extracted hot spots. To further improve the networks, deep supervision (DSV) and residual learning technologies were introduced.

**Results:**

We evaluated the performance of the proposed system using 246 bone scintigrams of prostate cancer in terms of accuracy of skeleton segmentation, hot spot extraction, and BSI measurement, as well as computational cost. In a threefold cross-validation experiment, the best performance was achieved by BtrflyNet with DSV for skeleton segmentation and BtrflyNet with residual blocks. The cross-correlation between the measured and true BSI was 0.9337, and the computational time for a case was 112.0 s.

**Conclusion:**

We proposed a deep learning-based BSI measurement system for a whole-body bone scintigram and proved its effectiveness by threefold cross-validation study using 246 whole-body bone scintigrams. The automatically measured BSI and computational time for a case are deemed clinically acceptable and reliable.

## Introduction

Radionuclide imaging is a useful means of examining patients who may have metastasis of the prostate, breast or lung cancers, which are common cancers globally [1, 2]. A typical screening method is bone scintigraphy, which uses Tc-99 m-methylene diphosphonate (MDP) [3] or Tc-99 *m*-hydroxymethylene diphosphonate (HMDP) [4] agents. Because visual interpretation of the bone scintigram lacks quantitative and reproducible diagnosis, quantitative indices have been proposed. Soloway et al. [5] proposed the extent of disease (EOD), which categorises bone scan examinations into five grades based on the number of bone metastases. It is simple but not suitable for detailed diagnosis. Erdi et al. [6] proposed the bone scan index (BSI), which standardises the assessment of bone scans [7], and they presented a region growing-based semiautomated bone metastatic lesion extraction method to measure the BSI. However, the method is time-consuming and less reproducible because seed regions must be manually inputted.

Yin et al. [8] proposed a lesion extraction algorithm using the characteristic point-based fuzzy inference system. Huang et al. [9] presented a bone scintigram segmentation algorithm followed by lesion extraction using adaptive thresholding with different cut-offs in different segmented regions. An alternative approach for lesion extraction was proposed by Shiraishi et al. [10], who presented a temporal subtraction-based interval change detection algorithm. Sajn et al. [11] proposed a classification method to classify a bone scan examination into *no pathology* or *pathology* using support vector machine with features derived from segmented bones. Sadik et al. [12–14] presented several algorithms which addressed skeleton segmentation, hot spot detection and classification of bone scan examinations. The algorithms in [12] were improved, where an active shape model (ASM) was employed for skeleton segmentation and an ensemble of three-layer perceptrons was introduced for hot spot detection [13], whose performance was evaluated with 35 physicians in [14].

It should be noted that the aforementioned studies [8–14] conducted hot spot detection and bone scan classification but did not assess BSI. One of the possible reasons for this might be low accuracy in the automated skeleton segmentation. For example, previous studies [8, 9] outputted polygonal regions, which roughly approximated bone regions. Although the skeleton segmentation performance in the previous study [12] was improved [13] by the use of ASM, it was found to be sensitive to the initial position of the model and image noise. In addition, the whole skeleton could be divided into only four parts, each of which included several different bones. This type of approximation will degrade the accuracy of the measured BSI because coefficients as given in the ICRP publication [15] used in the measurement differ in bones.

Some of the aforementioned problems have been solved using the atlas-based approach [16], in which a manually segmented atlas consisting of more than ten bones was nonlinearly registered to an input image, and labels in the deformed atlas were transferred to the image. The atlas-based approach was also employed in other studies [17–23], as were the commercialised computer-aided interpretation systems EXINIbone (EXINI Diagnostics AB, Lund, Sweden) and BONENAVI (FUJIFILM Toyama Chemical Co., Ltd., Tokyo, Japan). Accurate skeleton segmentation allows precise measurement of BSI [18] and accurate classification of bone scintigrams [17, 19–21]. Ulmet et al. [18] reported that the correlation between manual and automated BSI was 0.80 using EXINIbone. Horikoshi et al. [17] and Koizumi et al. [21] evaluated the performance of BONENAVI and Pertersen et al. [20] explored the performance of EXINIbone to demonstrate their effectiveness. Nakajima et al. [19] compared EXINIbone and BONENAVI using a Japanese multi-centre database. Brown et al. [22, 23] employed an atlas-based anatomical segmentation and proposed a new biomarker used in the commercially available system (MedQIA, Los Angeles, USA). The atlas-based segmentation is a promising approach but suffers from the problems of initial positioning of the atlas and differences in shape, direction and size between the atlas and skeleton of an input image. These problems might be solved by a multi-atlas-based approach [24]. However, it is a time-consuming process, which is not acceptable for clinical use.

Deep learning-based approaches have recently emerged in the field of medical image analysis [25]. This was initiated by the great success of an image recognition competition [26]. Numerous novel technologies [27–32] have been reported. For example, U-Net-type fully convolutional networks [28, 29] are some of the most successful networks for medical image segmentation, which might be useful for skeleton segmentation and extraction of hot spots of bone metastatic lesion.

This study presents a system consisting of skeleton segmentation and extraction of hot spots of bone metastatic lesion followed by BSI measurement. We employed a deep learning-based approach to achieve high accuracy in skeleton segmentation and hot spot extraction. One of the reasons for the low accuracy of skeleton segmentation and hot spot extraction in existing studies [6, 8, 14, 16–21] may be that anterior and posterior images have been independently processed, thus resulting in the inconsistent results. We used a butterfly-type network (BtrflyNet) [30] which fuses two U-Nets into a single network which can process anterior and posterior images simultaneously. Because a deep and complicated network might be problematic for the training process, we introduced deep supervision (DSV) [31] and residual learning [32], both of which are effective at avoiding gradients vanishing or exploding during the training of a deep network. We conducted the experiment using 246 cases of prostate cancer and demonstrated the effectiveness of the proposed system by comparing it with conventional approaches, namely multi-atlas-based skeleton segmentation and U-Net-based hot spot extraction.

## Methods

### Bone scintigraphy

**Fig. 1:**
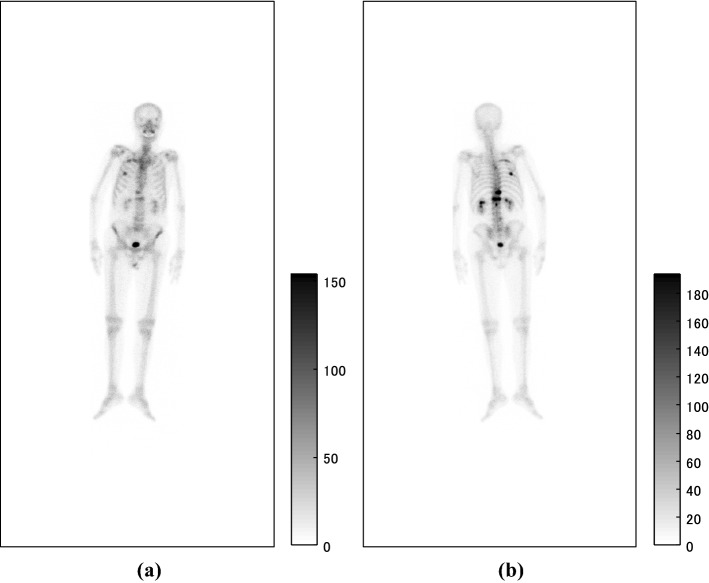
Pair of input **a** anterior and **b** posterior images

Inputs of the proposed system were anterior and posterior bone scintigrams as shown in Fig. 1, the sizes of which were 512 × 1024 pixels. The imaging systems were ‘VERTEX PLUS, ADAC’, ‘FORTE, ADAC’ and ‘BRIGHTVIEW X, Philips’ equipped with collimators named ‘VXGP’, ‘LEHR’ and ‘LEHR’, respectively. The energy peak was centred at 140 keV with a 10% window. The whole body was scanned for approximately ten minutes about 3 h after the intravenous injection of Tc-99 *m*-HMDP (555–740 MBq, Nihon Medi-Physics Co., Ltd, Tokyo, Japan), and the scan speed was 20 cm/min.

### Outline of skeleton segmentation

**Fig. 2:**
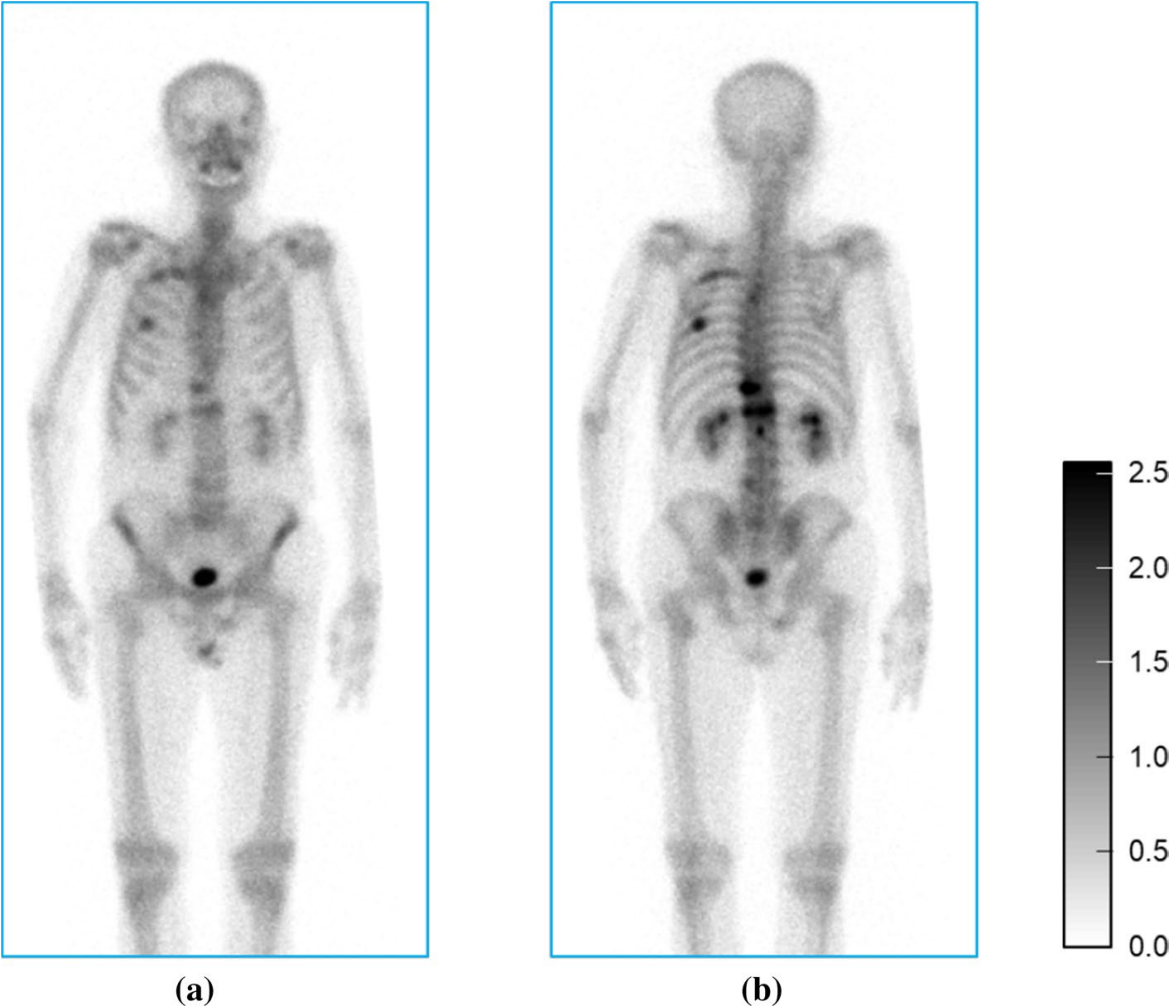
Spatially standardised **a** anterior and **b** posterior images with normalised grey values from Fig. 1 for skeleton segmentation

First, the posterior image was flipped horizontally and aligned to the anterior image for simultaneous segmentation. Second, spatial standardisation consisting of rotation, scaling and translation was applied to both the anterior and posterior images to ensure the body axis was parallel to a vertical axis of the image. The length from the top of head to the tip of the toe was 2000 mm. Third, grey-scale normalisation was performed for both images independently using the following equation.

1 $$ I_{\text{normalized}} = \left\{ {\begin{array}{*{20}l} {\log_{e} \left( {\phi \cdot \frac{{I_{\text{in}} - I_{98\% } }}{{I_{10\% } - I_{98\% } }} + 1} \right);} \hfill & {I_{\text{in}} > I_{98\% } } \hfill \\ {0;} \hfill & {\text{elsewhere}} \hfill \\ \end{array} } \right. $$

where $I_{\text{in}}$ is an input grey value, $I_{x\% }$ is the upper $x$th percentile and $\phi$ is the golden ratio. Central regions of the images (Fig. 2) were then forwarded to the trained BtrflyNet. Inverse transformation of the spatial standardisation and the alignment of the posterior image were performed to transfer the segmentation labels to the input images.

### Outline of hot spot extraction

**Fig. 3:**
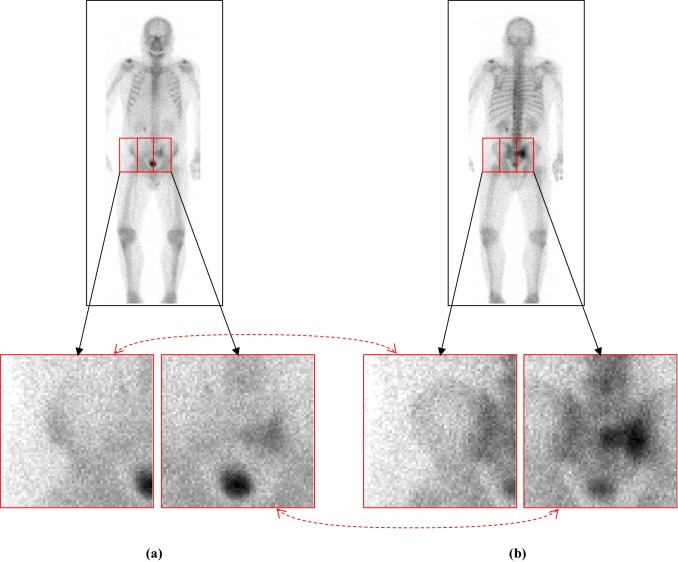
Pair of **a** anterior and **b** posterior patches with normalised grey values. Dotted red arrows indicate the correspondence between the two patches

First, a mask of a human was generated by applying a 3 $\times$ 3 pixel median filter and thresholding (1: I $\ge$ 4, 0: else) followed by opening and closing operations. (The structural element was a circle with radius of 2 pixel.) Second, grey-scale normalisation and registration between posterior and anterior images were conducted, both of which were the same as those in the skeleton segmentation. The image was then evenly divided into patch images of 64 $\times$ 64 pixel at every 32-pixel interval (Fig. 3).

Patch images that contained one or more pixels in the human mask were forwarded to the trained BtrflyNet for hot spot extraction. Finally, patch images with the extracted hot spots were integrated into an output image whose size was equal to that of the input image.

### BtrflyNets

**Fig. 4:**
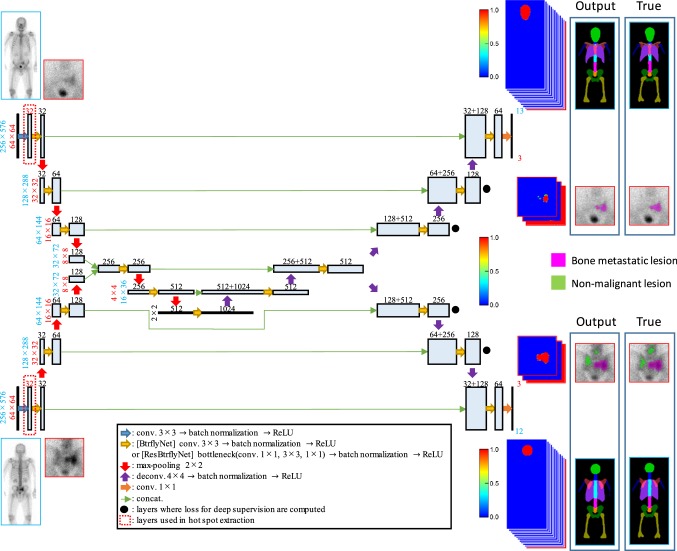
BtrflyNet for skeleton segmentation and hot spot extraction. Parameters of the network are listed, where blue numbers denote skeleton segmentation, red numbers indicate hot spot extraction and black numbers are common parameters for both networks. Note that the sizes of feature maps for the decoder part of the BtrflyNet are the same as those of the encoder part

BtrflyNets for skeleton segmentation and hot spot extraction are different networks but are nonetheless similar. Major differences exist in terms of the sizes of input and output images as well as the number of output layers. Skeleton segmentation input was a pair of anterior and posterior images of a whole body, and hot spot extraction input was a pair of anterior and posterior patch images. Output of anterior skeleton consisted of 13 layers corresponding to 12 bones (skull, cervical vertebrae, thoracic vertebrae, lumbar vertebrae, sacrum, pelvis, ribs, scapula, humerus, femur, sternum and clavicle) and background. Outputs of posterior skeleton were 12 layers for ten bones (skull, cervical vertebrae, thoracic vertebrae, lumbar vertebrae, sacrum, pelvis, rib, scapula, humerus and femur) and background. Note that one output layer in the posterior was for overlapped regions of the rib and scapula. Output for hot spot extraction was consisted of three layers each of which corresponded to a hot spot of bone metastatic lesion, hot spot of non-malignant lesion (e.g., fracture, infection) and others (e.g., physiological renal uptake, radioactive isotope distribution of bladder and background). In addition, sizes of feature maps of the BtrflyNets were different because of the size differences of input images. In Fig. 4, numbers of output layers and the sizes of feature maps are shown in blue for skeleton segmentation and in red for hot spot extraction. Furthermore, the BtrflyNet for hot spot extraction had an additional layer following the input layer enclosed by dotted red squares. This additional layer derives from improvement by residual blocks [32] which is described later.

### Loss functions

The loss functions to be minimised in the training of skeleton segmentation and hot spot extraction are given as follows.

#### Skeleton segmentation

2 $$ \left[ {\text{Generalised Dice loss}} \right]\quad {\mathcal{L}}_{\text{GDL}} = 1 - \frac{2}{C}\mathop \sum \limits_{c}^{C} \left( {\frac{{\mathop \sum \nolimits_{n}^{N} p_{cn} t_{cn} + \varepsilon }}{{\mathop \sum \nolimits_{n}^{N} p_{cn} + \mathop \sum \nolimits_{n}^{N} t_{cn} + \varepsilon }}} \right) $$

where *n* and *c* are indices of pixel and class (= bone metastatic lesion, non-malignant lesion and others) and *N* and *C* are total numbers of pixels and classes, respectively. In addition, $p_{cn}$ is the softmax of output $y_{cn}$ of the network and $t_{cn}$ denotes the true label in which the pixel value of the organ of interest is 1 and other is 0. Finally, $\varepsilon$ is a tiny value to prevent zero division.

3 $$ p_{cn} = {\text{softmax}}\left( {y_{cn} } \right) = \frac{{e^{{y_{cn} }} }}{{\mathop \sum \nolimits_{c}^{C} e^{{y_{cn} }} }} $$

#### Hot spot extraction

4 $$ \left[ {\text{Class weighted softmax cross entropy}} \right]\quad L_{\text{WSCE}} = - \frac{1}{N}\mathop \sum \limits_{n}^{N} \mathop \sum \limits_{c}^{C} w_{c} t_{cn} { \log }\left( {p_{cn} } \right) $$

where $w_{c}$ is a weight of class *c* to reduce the influence by the difference in the number of pixels.

5 $$ w_{c} = \frac{{N - \mathop \sum \nolimits_{n}^{N} t_{cn} }}{{N}} $$

### Improvements of networks

DSV [31] is introduced for skeleton segmentation, in which loss functions are computed at not only output layers but also at four layers neighbouring to output layers and as indicated by black dots in Fig. 4. Loss is a summation of the generalised Dice losses at the six layers.

Residual blocks [32] are used instead of convolutions and deconvolutions in the BtrflyNet for hot spot extraction. The improved BtrflyNet is called ResBtrflyNet in this study.

### Outputs of the system

The proposed system outputs segmented bones and detected hot spots, all of which are determined by using probability $p_{cn}$ in output layers of the trained BtrflyNets. The skeleton segmentation selects labels with a maximum $p_{cn}$ at each pixel. The hot spot extraction employs the threshold value of the following equation so that the sensitivity per hot spot of bone metastatic lesion is 0.9.

6 $$ \left\{ {\begin{array}{*{20}l} {\begin{array}{*{20}l} {p_{{{\text{meta}} .,n}} \ge th \to {\text{Hot spot by bone metastatic lesion}}} \hfill \\ {{\text{else if}} p_{{{\text{non-mal}} .,n}} \ge p_{{{\text{others}},n}} \to {\text{Hot spot by non-malignant lesion}}} \hfill \\ \end{array} } \hfill \\ {{\text{else}} \to {\text{others}}} \hfill \\ \end{array} } \right. $$

where $p_{cn}$ at the overlapped area of neighbouring patch images is computed by averaging $y_{cn}$ of the two patches.

The BSI is measured using segmented bones and extracted hot spots of bone metastatic lesions [18]. First, the correspondence between the bones and hot spots is determined. Second, a ratio between the area of the extracted hot spots and that of the corresponding bone is measured, and the weight fraction constant as given in the ICRP publication [15] is multiplied with the ratio. Finally, a summation of all values is outputted as BSI.

## Experimental set-up

The experiment was approved by the Ethics Committee at Osaka City University (Approval No. 3831) and Tokyo University of Agriculture and Technology (Approval No.30-30, 30-43). The total number of bone scintigrams was 246, derived from Japanese males with prostate cancer whose ages were from 52 to 95 (average: 72.8, standard deviation: 6.96). The dataset was divided into three groups to conduct threefold cross-validation. We also prepared a validation dataset to determine an optimal training iteration to avoid overtraining. In summary, 164 scans were for training, 41 for validation and 41 for testing. Because the validation and testing datasets were switched in onefold, we obtained test results from 246 total scans. The number of anterior and posterior patch pairs for onefold in training the hot spot extraction network was approximately 0.7 million.

### Initialisation and optimisation of the networks

In the training process, He’s initialisation [33] was used to initialise all weights of the networks. The loss functions were minimised using adaptive moment estimation (Adam) [34]. The detailed parameters are given as follows.

#### Skeleton segmentation

The parameters of Adam were set to *α* = 0.001, *β* = 0.9, *γ* = 0.999 and *ε* = 10^−8^. Note that *α* was decreased by one-tenth at the 1350th iteration in which the batch size was 6. The maximum number of iterations was set to 1620, and the optimal number of iterations was determined when the average Dice score from (7) of the validation dataset reached the maximum. The tiny value of *ε* from (2) was set to 0.001.

#### Hot spot extraction

The parameters of Adam were set to *α* = 0.001, *β* = 0.9, *γ* = 0.999 and *ε* = 10^−8^, where the batch size was 256. Augmentation was conducted by flipping an input image horizontally with a probability of 0.5. The maximum number of iterations was set to 50,000, and the optimal number of iterations was determined when the total number of misclassified pixels of the validation dataset was at the minimum.

### Performance evaluation

The Dice score between the segmented bone region and true region was computed to evaluate the performance of skeleton segmentation.

7 $$ {\text{Dice score}} = \frac{{2 \times \# \left( {{\text{``segmented bone region''}} \cap {\text{``true bone region''}}} \right)}}{{\# \left( {{\text{``segmented bone region''}} + \# {\text{``true bone region''}}} \right)}} $$

where $\# \left( {\text{region}} \right)$ denotes the number of pixels in the region. The sensitivity of hot spot detection, the numbers of false positive pixels and regions (8-connectivity) were used to evaluate hot spot extraction. Note that true regions of bones and hot spots were manually delineated by medical engineers and approved by medical doctors at the Department of Nuclear Medicine at the university hospital.

## Results

### Skeleton segmentation

**Fig. 5:**
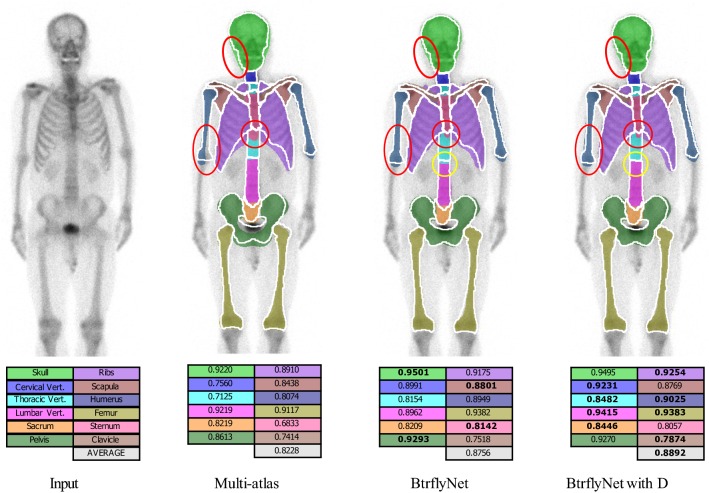
Typical results of skeleton segmentation. White lines and coloured regions are boundaries of true and segmented bone regions, respectively. Numbers denote Dice scores of bones in which the highest Dice scores among the three methods are bolded

**Fig. 6:**
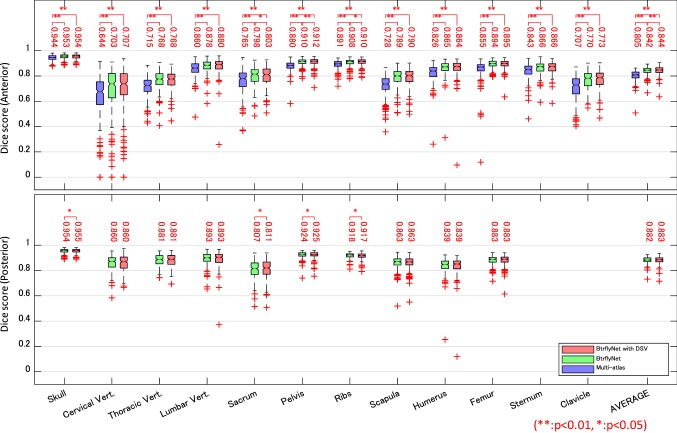
Dice scores of skeleton segmentation. Numbers indicate the median of scores. Statistical test was conducted by a Wilcoxon signed-rank test with the null hypothesis of ‘there is no difference in performance between the two methods’

Figure 5 presents the typical results of skeleton segmentation, and Fig. 6 shows Dice scores for all test cases. Note that the multi-atlas-based approach [24] employed B-spline-based non-rigid registration of 164 atlases from the training dataset and only anterior images were segmented because of high computational cost.

### Hot spot extraction

**Fig. 7:**
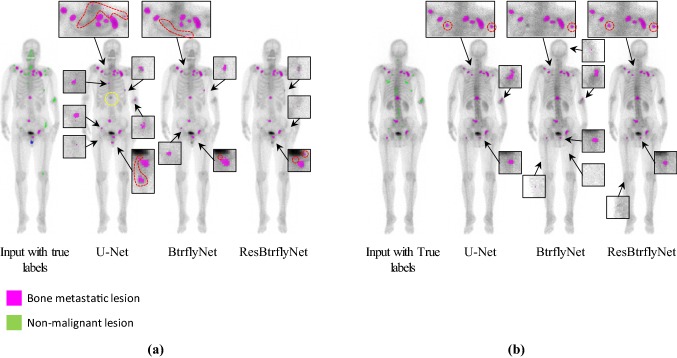
Typical extraction results of hot spots of bone metastatic lesions in **a** anterior and **b** flipped posterior images. False positives close to true bone metastatic lesions are circled by red dots, and a false negative is circled by yellow dots

Figure 7 shows the typical extraction results of hot spots of bone metastatic lesions when the sensitivity per hot spot of bone metastatic lesion was 0.9. Table 1 presents the number of false positive pixels, false positive regions and misclassified pixels by U-Net, BtrflyNet and ResBtrflyNet.

**Table 1:**
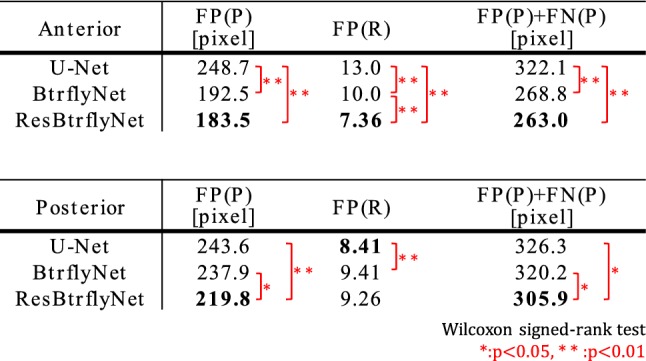
Average number of false positive pixels, false positive regions (8-connectivity) and misclassified pixels ({“false positives”} ∪ {“false negatives”}) when sensitivity per hot spot of bone metastatic lesion was 0.9

### Measurement of BSI

**Fig. 8:**
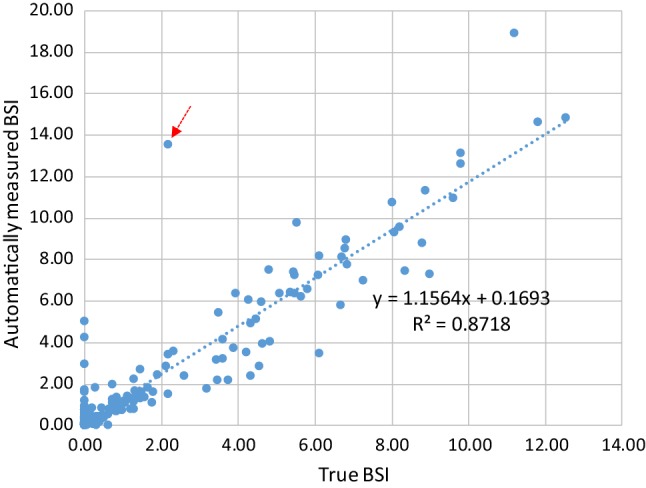
Relationship between automatically measured BSI and true BSI

Figure 8 compares automatically measured BSI with true BSI, which was computed using true regions of bones and hot spots of bone metastatic lesions.

### Computational cost

Average computation time for each test case was measured using a computer with 24 threads based on 41 cases for skeleton segmentation and five cases for hot spot extraction. The computer specifications were: OS: Ubuntu 16.04, CPU: Xeon Silver 4116. 12 Cores, 24 Threads, 2.10 GHz $\times$ 2, Memory: 196 GB.

#### Skeleton segmentation (without pre- and post-processes)

- Multi-atlas (anterior image only) = 5287 s.
- BtrflyNet (or BtrflyNet with DSV) = 16 s.

#### Hot spot extraction

- U-Nets = 70 s.
- BtrflyNet = 63 s.
- ResBtrflyNet = 94 s.

## Discussion

### Skeleton segmentation

The red circles in Fig. 5 show typical errors in segmentation by the multi-atlas-based method because of atypical shapes or directions of the skull, right humerus and sternum. Figure 6 suggests that the multi-atlas-based method was inferior to BtrflyNet-based approaches for all organs, and the differences were statistically significant.

**Fig. 9:**
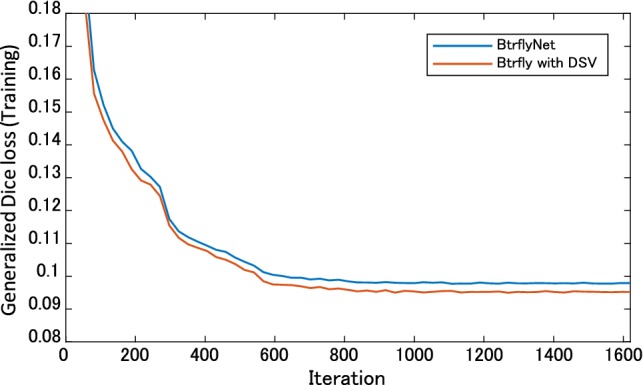
Transitions of generalised Dice loss in the training

An example of the improvement gained by DSV is indicated by the yellow circle in Fig. 5. Figure 6 indicates that BtrflyNet with DSV was superior to the naïve BtrflyNet for nine out of 12 bones in an anterior image and three out of ten bones in a posterior image. Statistical differences were observed for three bones each in anterior and posterior images. By contrast, the rib in a posterior image was the only bone for which the naïve BtrflyNet was statistically superior. Therefore, we concluded that BtrflyNet with DSV was the best in our experiment. The main reason may have been lower loss during training. Figure 9 shows transitions of training losses at the output layers, where the red line of the BtrflyNet with DSV was lower than the blue line of the naïve BtrflyNet, which suggests that the DSV was effective at reducing loss in the training data.

### Hot spot extraction

In the anterior image of Fig. 7, U-Net failed to detect one hot spot by a bone metastatic lesion, and 17 false positive regions existed, whereas BtrflyNet and ResBtrflyNet detected all hot spots of bone metastatic lesions, where the numbers of false positive regions were seven and five, respectively. In addition, BtrflyNet and ResBtrflyNet showed high consistency between the results of anterior and posterior images. The difference in the number of false positive regions by BtrflyNets and ResBtrflyNet was one, whereas that by U-Nets was 13. This fact suggests that simultaneous process of both images by BtrflyNet and ResBtrflyNet realised a high consistency, thus leading to high performance.

**Fig. 10:**
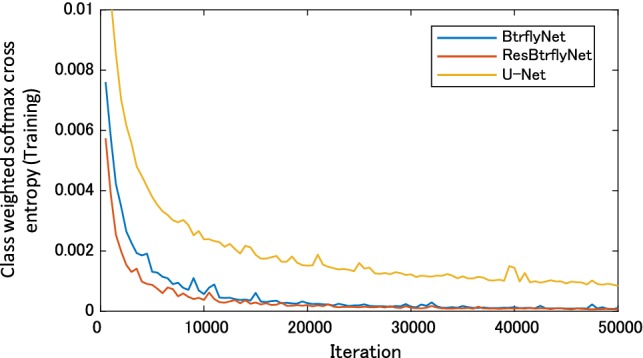
Transitions of class weighted softmax cross-entropy during the training

Table 1 suggests that ResBtrflyNet was the best in terms of the numbers of false positive and misclassified pixels as well as number of false positive regions in an anterior image. The differences among the networks could be because of the difference in losses of the training dataset (Fig. 10), in which the loss of ResBtrflyNet was the minimum. In fact, the loss of ResBtrflyNet at the optimal number of iterations was 29.9% lower than that of BtrflyNet.

### BSI measurement

Figure 8 shows good correlation between the automatically measured BSI and true BSI when using the best combination of networks with the highest performance, namely the BtrflyNet with DSV for skeleton segmentation and ResBtrflyNet for hot spot extraction. The cross-correlation was 0.9337, which was higher than 0.80 as reported by Ulmert et al. [18] and seems reliable for clinical use. Note that comparing the two values directly is difficult because the dataset used was different. However, the higher cross-correlation suggests a promising performance with the proposed system.

**Fig. 11:**
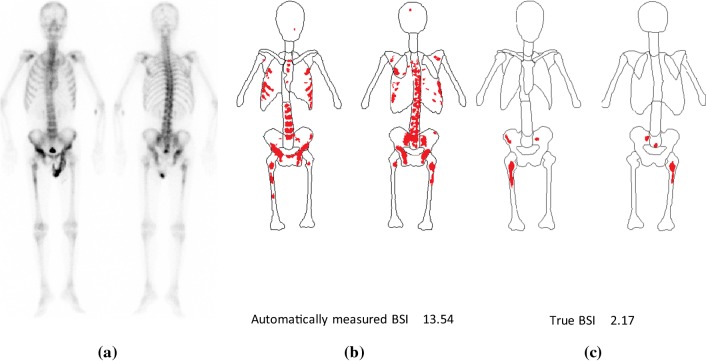
Case with the maximum error with the BSI measurement: **a** pair of anterior and posterior images. **b** Automatically recognised results and **c** true region of skeleton and hot spots of bone metastatic lesions

The limitations of the proposed system must be mentioned. Figure 11 shows the case with the maximum error with the BSI measurement (red arrow in Fig. 8). Although the skeleton was recognised correctly, hot spots by osteoarthritis in thoracic and lumbar vertebrae were misclassified as hot spots of bone metastatic lesions. One possible reason for this failure is the limited amount of training data for osteoarthritis. Training using a large dataset with osteoarthritis cases remains an important future study.

### Computational cost

The proposed BtrflyNet-based skeleton segmentation took 16 s. for the case using 24 threads. By contrast, the cost of the multi-atlas-based method for an anterior image was over 300 times greater than that of BtrflyNet. The most time-consuming step was non-rigid registration, which took 3420 s. on average, even when ten registration processes ran in parallel.

In the hot spot extraction experiments with multiple threads, the naïve BtrflyNet was the fastest because it shared the deepest layers for anterior and posterior images as compared with U-Net. ResBtrflyNet was 1.5 times longer than the naïve BtrflyNet because of the high computational cost of residual blocks. However, the difference was not considerable.

The cost of the best combination of networks including pre- and post-processes (e.g., spatial standardisation) was 112.0 s. per case, which seems acceptable for clinical use.

## Conclusion

This study proposed a deep learning-based image interpretation system for automated BSI measurements from a whole-body bone scintigram, in which BtrflyNets were used to segment the skeleton and extract hot spots of bone metastatic lesions. We conducted threefold cross-validation using 246 bone scintigrams of prostate cancer to evaluate the performance of the system. The experimental results revealed that the best performance was achieved by a combination of BtrflyNet with DSV for skeleton segmentation and BtrflyNet with residual blocks, and the number of misclassified pixels for which was minimum. The computational time of both processes for a case was 112.0 s., and automatically measured BSI showed high correlation (0.9337) with the true BSI, both of which is deemed clinically acceptable and reliable.

An important future work will involve increasing the size of the training dataset to improve the misclassification of the osteoarthritis case. The effect of dataset size on performance would be an interesting topic. Optimising the hyper-parameters of deep networks, e.g., number of layers, number of channels (feature maps) and weights in loss functions, is also essential to boost the performance in terms of segmentation and extraction accuracy as well as computational cost. It would be interesting to perform a leave-one-out examination for further performance analysis. Developing an anatomically constrained network is also necessary to avoid anatomically the wrong results and to enhance the reliability of the system.
