# Correction to: Automated measurement of bone scan index from a whole-body bone scintigram

**DOI:** 10.1007/s11548-020-02119-w

**Published:** 2020-02-01

**Authors:** Akinobu Shimizu, Hayato Wakabayashi, Takumi Kanamori, Atsushi Saito, Kazuhiro Nishikawa, Hiromitsu Daisaki, Shigeaki Higashiyama, Joji Kawabe

**Affiliations:** 1 Institute of Engineering, Tokyo University of Agriculture and Technology, 2-24-16 Naka-cho Koganei, Tokyo, 184-0012 Japan; 2 Nihon Medi-Physics Co., Ltd, 3-4-10 Shinsuna Koto-ku, Tokyo, 136-0075 Japan; 3 Department of Radiological Technology, Gunma Prefectural College of Health Sciences, 323-1 Kamioki-machi Maebashi, Gunma, 371-0052 Japan; 4 Department of Nuclear Medicine, Graduate School of Medicine, Osaka City University, 1-4-3 Asahimachi Abeno-ku, Osaka, 545-8585 Japan

## Correction to: International Journal of Computer Assisted Radiology and Surgery 10.1007/s11548-019-02105-x

The article Automated measurement of bone scan index from a whole-body bone scintigram, written by Akinobu Shimizu, Hayato Wakabayashi, Takumi Kanamori, Atsushi Saito, Kazuhiro Nishikawa, Hiromitsu Daisaki, Shigeaki Higashiyama and Joji Kawabe, was originally published electronically on the publisher’s Internet portal on 13 December 2019 without open access. With the author(s)’ decision to opt for Open Choice, the copyright of the article changed on 16 January 2020 to © The Author(s) 2020 and the article is forthwith distributed under a Creative Commons Attribution 4.0 International License (https://creativecommons.org/licenses/by/4.0/), which permits use, sharing, adaptation, distribution and reproduction in any medium or format, as long as you give appropriate credit to the original author(s) and the source, provide a link to the Creative Commons license and indicate if changes were made.

